# Is serum‐derived exosomal hTERT transcript a marker of oncogenic activity in primary brain tumors? An exploratory study

**DOI:** 10.1002/cam4.6784

**Published:** 2023-12-28

**Authors:** Orit Uziel, Andrew A. Kanner, Einat Beery, Sapir Lev, Meir Lahav, Suzana Horn‐Fichman, Sagi Har Nof, Yuseph Laviv, S. Yust‐Katz, Alexandra Amiel, Ramez Abu Shkara, Mustafa Siddeeq, Adva Levy‐Barda, Pia Raanani, Yaron Sela, Zvi Cohen, Tali Siegal

**Affiliations:** ^1^ The Felsenstein Medical Research Center Petah Tikva Israel; ^2^ Institute of Hematology Davidoff Cancer Center, Rabin Medical Center Petah Tikva Israel; ^3^ Sackler School of Medicine Tel‐Aviv University Tel Aviv Israel; ^4^ Department of Neurosurgery Rabin Medical Center Petah Tikva Israel; ^5^ Neuropathology, Department of Pathology Rabin Medical Center Petah Tikva Israel; ^6^ Neurooncology Unit Davidoff Cancer Center, Rabin Medical Center Petah Tikva Israel; ^7^ Department of Neurosurgery Sheba Medical Center Ramat‐Gan Israel; ^8^ Biobank, Department of Pathology Rabin Medical Center Petah Tikva Israel; ^9^ The Center of Internet Psychology Reichman University Herzliya Israel; ^10^ Hebrew University and Medical School Jerusalem Israel

**Keywords:** atypical meningioma, exosomes, glioblastoma, liquid biopsy, meningioma, telomerase

## Abstract

**Background:**

In order to proliferate indefinitely, all tumors require a telomere maintenance mechanism. The expression of human telomerase reverse transcriptase (hTERT) enables telomere maintenance and provides cancer cells with limitless replicative potential. As such, it may serve as an attractive biomarker for oncogenic activity. This study explored whether a liquid biopsy that analyses blood derived exosomal hTERT transcript (e‐hTERT‐trans) may serve as such a biomarker in gliomas and meningiomas when compared to healthy controls.

**Methods:**

Exosomes were isolated from the pre‐operative sera of patients' samples stored in the biobank of both Rabin and Sheba Medical Centers. The levels of e‐hTERT‐trans were measured in 81 healthy controls, 117 meningiomas, 17 low‐grade gliomas, and 61 glioblastomas. Clinical parameters of the patients were collected retrospectively and compared to the levels of the e‐hTERT‐trans.

**Results:**

The upper normal limit of controls e‐hTERT‐trans was 1.85 relative quantitation (RQ). The rate of detection increased with rising tumor grade and correlated with tumor recurrence in meningiomas: mean RQ without recurrence (2.17 ± 11.7) versus with recurrence (3.59 ± 4.42; *p* = 0.002). In glioblastomas, preoperative measurements correlated with tumor volume and with the disease course on serial sampling.

**Conclusions:**

We demonstrated for the first time that the expression of e‐hTERT‐trans transcript can be measured in the serum of primary brain tumors. This exosomal marker carries the potential to serve as a biomarker once used in conjunction with other clinical and radiological parameters. Future studies are required to investigate whether the sensitivity could be augmented and whether it can be implemented into routine patients care.

## Graphical abstract

Our study demonstrates for the first time that the expression of hTERT mRNA transcript can be measured in the serum of both meningioma and glioma patients. As it correlated with tumor recurrence in meningiomas and with tumor volume and the disease course in glioblastomas it carries the potential to serve as a biomarker once used in conjunction with other clinical and radiological parameters.

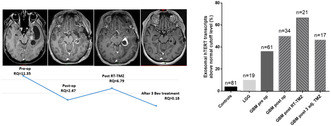

## 1 INTRODUCTION

A hallmark of a cancer cell is its ability to sustain proliferative signaling and replicate indefinitely.^1^ In normal cells the replicative potential is limited by the shortening of telomeres, the chromosome end parts, with each replicative cycle, eventually leading to cellular senescence. The addition of telomere sequences to these ends of chromosomes allows for successive replicative cycles providing limitless lifespan without chromosomal DNA shortening. The enzyme telomerase, an RNA‐dependent DNA polymerase, synthesizes telomeric DNA repeats to maintain telomere homeostasis, while human telomerase reverse transcriptase (hTERT) subunit of the enzyme is the rate‐limiting catalytic subunit of telomerase.^2^ As such, telomerase activity is downregulated in most normal cells by transcriptionally inactivation through epigenetic regulation of the hTERT promoter.^3^ In contrast, in stem cells and mainly in neoplastic tissues, telomerase activity is highly upregulated^4^ and thus counteracting the shortening of telomeres thus allowing for an unlimited replicative potential. Therefore, it is not surprising that telomerase upregulation is present in almost 90% of malignancies although the mechanisms of its activation and regulation varies and is not always completely understood.^5^, ^6^, ^7^, ^8^, ^9^ Most cancers demonstrate hTERT gene alterations including promoter mutation, gene translocations and DNA amplification.^10^ Overall, hTERT expression correlates closely with hTERT transcript levels, regardless of the underlying mechanism inducing its aberrant expression.^11^, ^12^ Considering the importance of telomerase activity to the perpetuation of malignant clones and the correlation between telomerase activity and its transcript levels, we selected the hTERT transcript levels as a potential biomarker for oncogenic activity.^13^ We assumed that measurement of circulating hTERT transcript can be used as a possible liquid biopsy based diagnostic marker in the clinical setting. In fact, we were able to show that blood derived exosomal hTERT transcript secretion may serve as a potential pan‐cancer marker in systemic solid and hematological malignancies.^13^ However, it has not been tested in brain tumors and it remains unclear whether circulating exosomal hTERT transcript reflects the oncogenic activity of central nervous system malignancies.

Liquid biopsies enable the minimally invasive collection and analysis of circulating biomarkers released from cancer cells and from other tissues, representing therefore a promising candidate for future integration in the current standard of care. Liquid biopsy has shown to be a powerful clinical tool for several cancers, including breast, bowel and lung cancer where it is emerging as important means for early detection and for monitoring therapy response mainly by detection of circulating tumor cells and circulating cell‐free nucleic acids (cDNA and cRNA).^14^, ^15^, ^16^ Despite the potential, the status of liquid biopsies in the field of brain tumors remains experimental and its clinical applicability either as a diagnostic or as a predictive tool remains ambiguous.^17^

Exosomes are nanovesicles (30–150 nm in diameter), secreted from virtually all cell types including cancer cells. They are found in biological fluids such as blood, urine, saliva, and cell culture media and carry a cargo of nucleic acids of all types, proteins and other molecules, reflecting the molecular makeup of their cells of origin.^18^, ^19^, ^20^ Since the molecular composition of exosomes reflects, at least partially, the physiological or pathophysiological makeup of their cells of origin, exosomes have a significant potential as a novel type of biomarkers. In this study we evaluated whether liquid biopsy which analyzes blood‐derived exosomal hTERT transcript secretion may serve as a potential biomarker for oncogenic activity in the most common brain tumors namely, gliomas, and meningiomas.

## 2 PATIENTS AND METHODS

### 2.1 Study population and sample collection

The study has been approved by the institutional review board and ethic committees of Rabin Medical Center, Petah Tikva, Israel and of Sheba Medical Center, Ramat Gan, Israel. We analyzed 368 serum samples for blood‐derived exosomal hTERT transcript. The serum samples were obtained from healthy control volunteers who signed an informed consent form, and all samples of brain tumor patients were obtained from the tumor biobanks of both institutions. The analyzed serum samples included 81 healthy controls, 81 preoperative glioblastomas (GBM) samples plus 70 serial GBM samples. In addition, 117 meningiomas and 19 low‐grade glial tumors (10‐astrocytomas world health organization [WHO] 2, 2‐pilocytic astrocytomas, 5‐oligodendrogliomas WHO 2, and 2‐ependymomas WHO 2) were analyzed (Table 1). The meningioma cohort included 80 WHO grade 1 meningiomas and 37 WHO grade 2 meningiomas. All brain tumors' serum samples were obtained prior to the surgical procedure on the day of surgery. Additional samples were drawn from GBM patients 3–4 weeks after surgery and prior to radiotherapy (34 patients), after completion of concurrent radiotherapy‐temozolomide course (21 patients) and after completion of three adjuvant cycles of temozolomide (17 patients) and served for evaluation of the dynamic changes of exosomal hTERT transcript. All follow‐up blood samples of GBM patients were obtained within 14 days of the most recent magnetic resonance imaging (MRI) study. Clinical, radiological, and histological data were collected from medical reports as well as from preoperative MRI. MRI images (using T1 contrast and FLAIR sequences) were analyzed for volumetric calculation by using a cranial navigation software (Brainlab iplan, Munich, Germany). Tumor and FLAIR hyperintensity volumes were calculated in cm^3^. Date of diagnosis and date of death or last follow‐up was available for GBM patients, and used for survival calculation. For the meningioma cohort data on tumor recurrence was collected for those patients who had a minimum follow‐up of 12 months. A shorter observation period was considered too short for evaluation of tumor recurrence. Baseline characteristics of the analyzed cohorts are presented in Tables 2 and 3.

**TABLE 1:** Preoperative telomerase transcript levels of the study cohorts.

	No. of patients	Telomerase transcript level				*p*‐value vs. controls
		Mean ± SD	Median	Range	Above normal cutoff	
Controls	81	0.50 ± 0.67	0.29	0–4.05	2.4%	
Meningioma WHO 1	80	1.09 ± 2.27	0.24	0–15.31	16.25%	0.831
Meningioma WHO 2	37	3.49 ± 16.33	0.20	0–99.66	18.9%	0.477
LGG	19	0.97 ± 0.6	0.16	0–1.92	10.5%	0.087
GBM	61	4.58 ± 11.97	0.99	0–86.52	36%	<0.001

Abbreviations: GBM, glioblastoma; LG, low‐grade glioma.

**TABLE 2:** Baseline characteristics of the meningioma cohort and telomerase transcript levels.

Clinical data of meningioma cohort		Telomerase transcript level	
		Mean ± SD	Median (range)
Age (median; range)	62 (21–82)		
Sex			
Female	78 (66.6%)	1.17 ± 2.75	0.16 (0–15.31)
Male	39 (33.6%)	2.23 ± 11.45	0.31 (0–99.66)
WHO grade 1	80 (68.3%)	1.02 ± 2.26	0.24 (0–15.31)
WHO grade 2	37 (31.6%)	3.74 ± 0.25	0.25 (0–99.66)
Location			
Convexity	37 (31.6%)	0.68 ± 1.25	0.23 (0–5.09)
Falx/Parasagital	32 (32%)	3.88 ± 17.52	0.18 (0–99.66)
Skull base	27 (23.1%)	1.09 ± 1.92	0.32 (0–7.60)
Posterior fossa	15 (12.8%)	2.02 ± 4.38	0.14 (0–5.54)
Others	6 (5.1%)	1.52 ± 2.06	0.71 (0–5.54)
Preoperative tumor volume (median; cm^3^)	23.91 (0.38–186)	37.65 ± 38.43	23.91 (0.38–186)
Median follow‐up (months)	14.7 (0–56)		
Tumor recurrence			
No	75 (63%)	2.17 ± 11.7	0.15 (0–99.6)
Yes	15 (12.6%)	3.59 ± 4.42**	2.56 (0–15.31)
Unknown/short F/U	27 (23.1%)	0.56 ± 0.81	0.27 (0–3.52)
Muscle/Bone/Fat involvement	17 (15.7%)	0.85 ± 1.83	0.20 (0–7.6)
Necrosis	28 (23.7%)	1.07 ± 1.61	0.33 (0–5.54)
Maximal Ki‐67 index (median, %); *n* = 87	4.51 (1–20%)	7.84 ± 4.51	4.51 (1.0–20)
Number of mitoses per 10HPF; *n* = 60	3 (0–14)	3.63 ± 2.85	3.0 (0–14)
*TERT* promoter status (grade 2 only)			
Wild type	20 (54%)		
Not done	17 (45.9%)		

Abbreviations: F/U, follow‐up; HPF, high‐power fields.

** *p* = 0.002.

**TABLE 3:** Baseline characteristics of the glioblastoma cohort and telomerase transcript levels.

Clinical data of glioblastoma cohort		Telomerase transcript level	
		Mean ± SD	Median (range)
Age (median; range)	64 (24–81)		
Sex			
Female	29 (47.5%)	2.23 ± 0.27	0.27 (0–99.66)
Male	32 (52.5%)	1.16 ± 0.16	0.16 (0–15.31)
IDH 1 WT	60 (98.3%)	4.70 ± 12.53	0.98 (0–86.52)
IDH 1 mutated	1 (1.6%)	13.11	
*TERT* promoter status			
Mutated	43/54 (79.6%)	12.87 ± 28.01	1.79 (0.04–86.52)
C228T	30/43 (69.7%)		
C250T	13/43 (30.2%)		
Wild type	11/54 (20.3%)	3.15 ± 5.89	0.91 (0–28.8)
Unknown	7/61 (11.5%)		
MGMT methylation status			
Unmethylated	20/39 (51.3%)	8.19 ± 19.42	1.27 (0–86.52)
Methylated	19/39 (48.7%)	2.84 ± 1.69	1.69 (0–13.11)
Unknown	22/61 (36%)	2.81 ± 6.19	0.95 (0–28.80)
Preop. enhancing tumor volume (median; cm^3^)	32.2 (0.83–107.6)		
Preop. FLAIR hyperintense volume (median; cm^3^)	90.3 (3.8–246.6)		
Preop. sample	61 (100%)	4.59 ± 11.97	0.99 (0–86.52)
Postop. and Pre‐RT‐TMZ sample	34/61 (55.7%)	3.95 ± 5.86	1.59 (0–27.08)
Post RT‐TMZ	21/61 (34.4%)	8.41 ± 14.37	4.32 (0.04–63.33)
Following 3 adjuvant TMZ cycles	17/61 (27.8%)	2.17 ± 3.07	0.92 (0–12.0)*

Abbreviations: IDH, isocitrate dehydrogenase; Postop, postoperative; Preop., preoperative; RT‐TMZ, concomitant radiotherapy and temozolomide; TMZ, temozolomide; WT, wild type.

* *p* = 0.06.

### 2.2 Exosomes purification

A 10 mL blood sample was obtained from each study participant (patients and healthy donors). Samples were centrifuged at 2500 RPM for 10 min half an hour after blood extraction to allow the clotting of the rest of the blood. Then the serum fraction was isolated by removing the upper liquid phase of the centrifuged sample. Sera samples were subsequently stored at −80°C until processed for exosome isolation. Exosomes were isolated from 2 mL sera samples by using the total exosome isolation kit (Invitrogen, CA, USA) according to the manufacturer's instructions. The purity of the exosomes was verified by the presence of specific exosomal marker (CD81) by flow cytometry. Additionally, exosomes' size and concentrations were analyzed in the NanoSight tracking device as previously described.^21^

### 2.3 RNA purification

RNA from exosomes was purified with the Total Exosome RNA and Protein Isolation Kit (Invitrogen, CA, USA) according to the provided manual.

### 2.4 cDNA synthesis

mRNA was reverse transcribed by using the High‐Capacity cDNA Reverse Transcription Kit (App. Bio., CA, USA) according to the manufacturer's instructions. By using this method the cDNA is formed only from the isolated mRNA and not from the other RNA species. As the specific Q‐RT PCR primers are directed around the exon‐exon junction, only mature RNA can be amplified by them, both of the GAPDH (the reference gene) and the hTERT (target gene).

### 2.5 hTERT expression by real‐time PCR

The expression of hTERT was measured relatively to that of the GAPDH as a reference gene. Gene amplification of the hTERT was executed using the following set of primers labeled with FAM (Applied Biosystems, CA, USA).

*hTERT*: Forward: 5'‐GTACTTTGTCAAGGTGGATGTGA‐3'

Reverse: 5'‐GCTGGAGGTCTGTCAAGGTAGAG‐3′

Primers for the amplification of the GAPDH gene were purchased from Thermo‐Fischer as a TaqMan on demand (#HS99999905).

PCR reactions were prepared with the Taqman fluorophore labeled primers (Applied Biosystems, CA, USA), run and analyzed on the Step One detection system (Applied Biosystems, CA, USA). Reactions were performed for 50 cycles; a normal value (no expression of hTERT) was arbitrarily defined as 1 for further calculation purposes. Values of each sample was calculated according to the formula CT^−ΔΔCT^. In order to detect the tiny amount of the transcript of hTERT in the isolated exosomes we performed a relatively high number of RT‐PCR cycles. The levels of the hTERT transcripts were normalized as described above to those of the GAPDH transcript as a reference gene.

### 2.6 Statistical analysis

The data presented in this study were analyzed using the SPSS software version 25. Descriptive statistics were performed using means and standard deviations for the continuous variables and frequencies for the discrete variables. The cutoff for hTERT transcript expression was calculated by exceeding the average value of the control group with additional two standard deviations. To correlate the expression of exosomal hTERT with the type of tumor and healthy controls, the Mann–Whitney test was conducted. Nonparametric tests were conducted to calculate associations between the expression of exosomal hTERT and clinical parameters, univariate comparisons were performed using the Mann–Whitney tests independent group comparisons for the continuous variables and the Wilcoxon tests for the dependent continuous variable comparisons. Bivariate comparisons between continuous variables were done using the Spearman's rho pairwise correlations. Wilcoxon signed rank test was carried out to compare the expression of exosomal hTERT transcript over four time points and time points 2, 3, and 4 were tested against the pre‐operative time point. Survival curves were generated using the Kaplan–Meier method to assess differences in survival between patients with high hTERT transcript values and patients with low exosomal hTERT expression; Significance for the *p*‐value was considered as lower than 5%.

## 3 RESULTS

### 3.1 Isolation of exosomes

**FIGURE 1:**
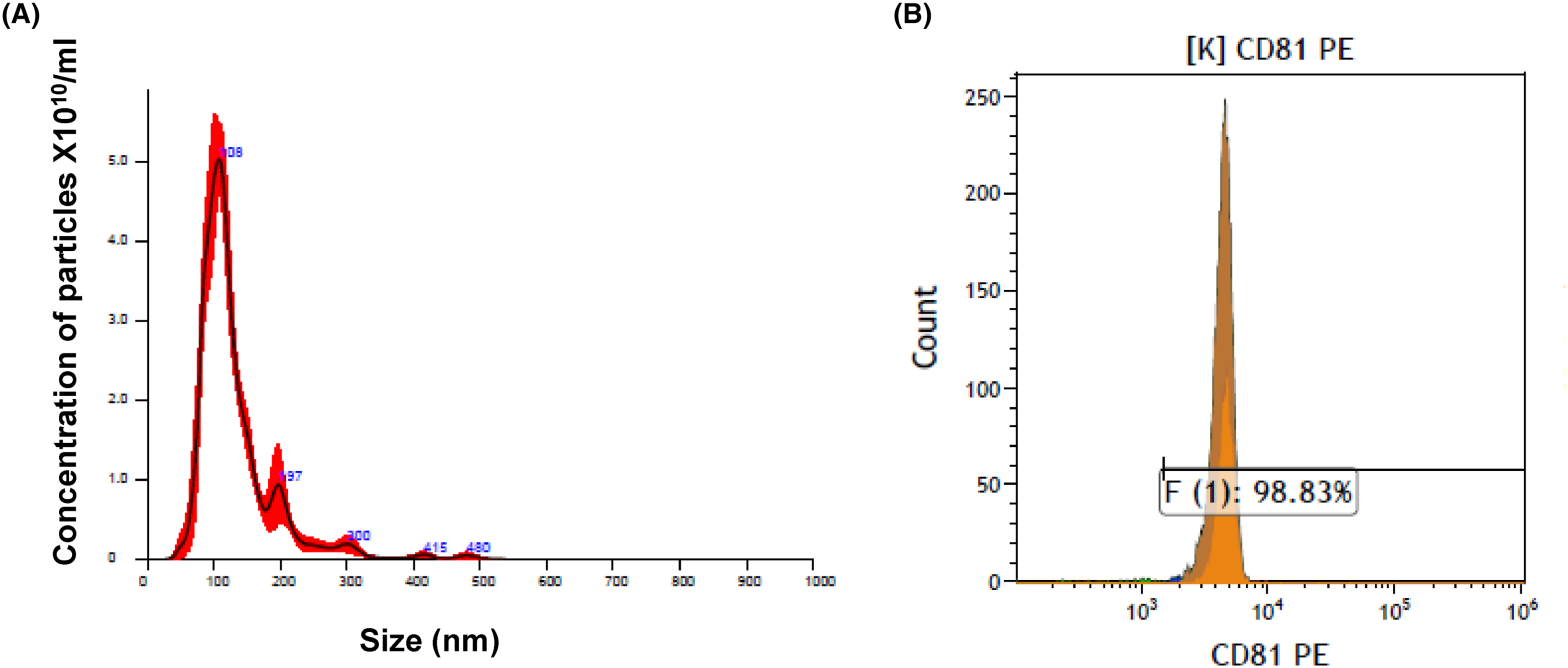
Characterization of serum derived exosomes. (A) Nano Sight Tracking analysis of exosomes isolated by a commercial kit: A typical representation of a nanoparticle tracking analysis with highest concentration of particles ranging in size between 50 and 200 nm. (B) Flow cytometer analysis of the expression of CD81 on the membrane of the isolated exosomes. Shown here is an example of the flow cytometer analysis.

Exosomes were isolated from the serum samples. We assessed the purity and concentration of exosomes by the Nano‐Sight tracking analysis (NTA) and by flow cytometry (Figure 1). NTA analysis identified the presence of a relatively large amount of 50–150 nm particles (Figure 1A) and flow cytometry demonstrated the presence of CD81 on these particles (Figure 1B). Together, these analyses verified that the isolated nanoparticles are exosomes.

### 3.2 Exosomal hTERT transcript levels

The mean level of hTERT transcripts of the 81 healthy controls was 0.50 ± 0.67 and the median value was 0.29 (range 0.0–4.05). The cutoff for elevated telomerase transcript level was set as the average value of the control group with additional 2 standard deviations, at a value of 1.85.

**FIGURE 2:**
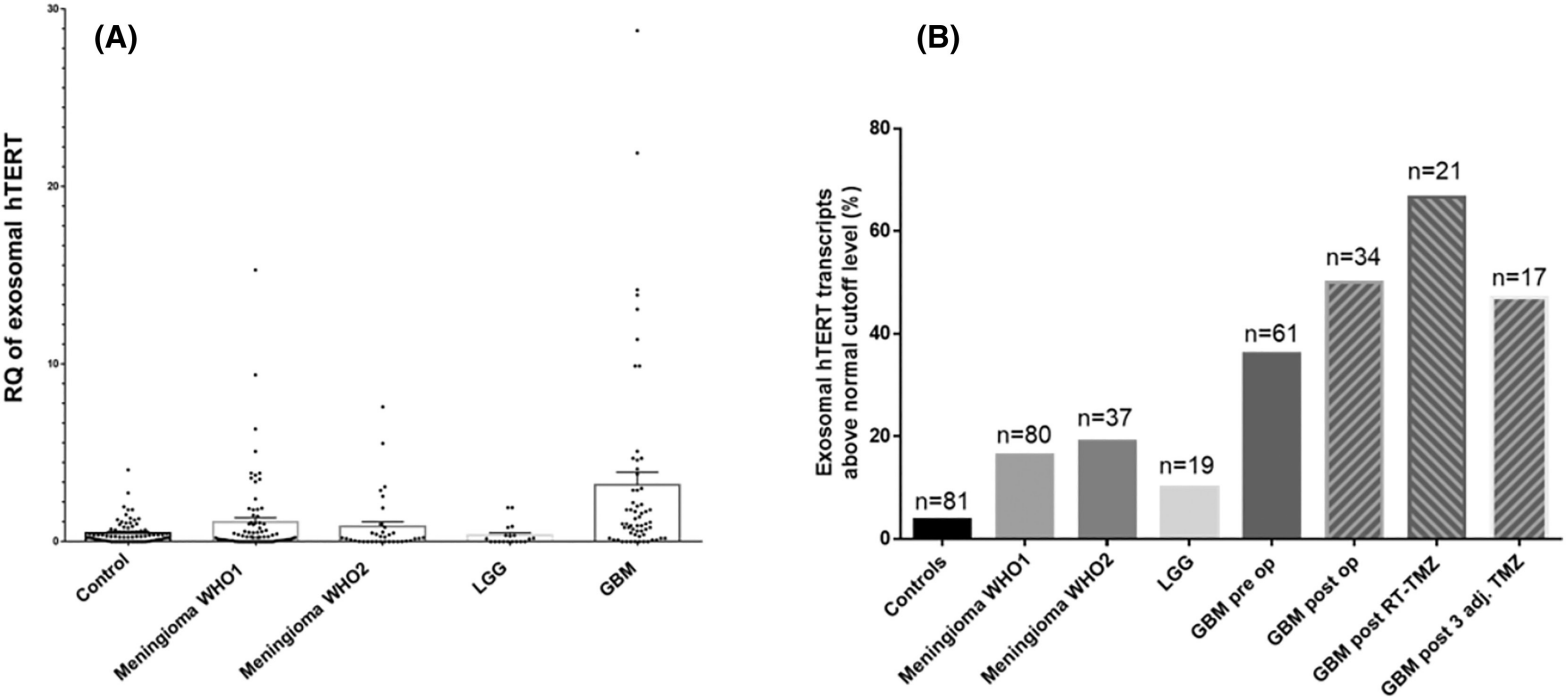
Exosomal hTERT transcript levels for all study cohorts. (A) Preoperative mean levels of the hTERT transcript. (B) The proportion of preoperative elevated hTERT transcript expression above normal cutoff level for all study cohorts, and overtime for glioblastoma cases. GBM‐glioblastoma; LGG‐ low‐grade gliomas; RT, radiotherapy; TMZ, temozolomide; Adj, adjuvant; EQ, relative quantitation of the hTERT transcript levels.

Table 1 and Figure 2 present the preoperative mean levels of the hTERT transcript for all study cohorts. The lower grade tumors namely, meningiomas (WHO grades 1 and 2) and low‐grade glial tumors did not differ significantly from the control group. Although the mean transcript value of the atypical meningioma cohort exceeds the cutoff value of the normal level. In contrast, the mean level of GBM patients (4.58 ± 11) significantly differed from the control group (*p* < 0.001). The proportion of high serum hTERT transcript levels was higher in all tumor cohorts' samples than in the control group (Table 1; Figure 2B) although statistically significant value was reached only for the GBM cohort. These results suggest that with increased oncogenic activity it becomes more likely to detect high levels of circulating exosomal hTERT transcript.

**FIGURE 3:**
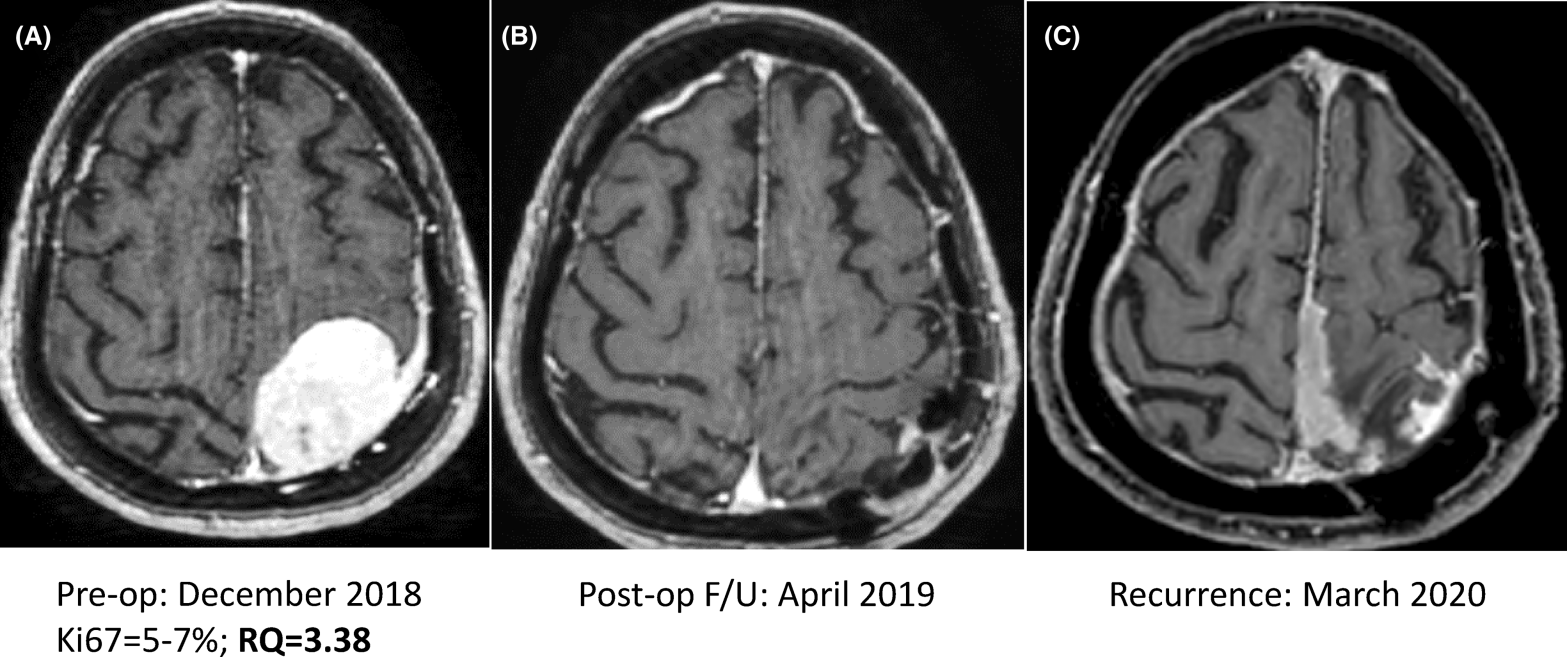
Gadolinium enhanced MRI studies of a 58‐year‐old female with WHO 1 meningioma with a preoperative elevated level of circulating exosomal hTERT transcripts. (A) Preoperative imaging. The serum relative quantification (RQ) value for telomerase expression prior to surgery was above normal level of healthy controls. (B) Postoperative follow‐up (F/U) imaging confirms gross total resection of the Grade 1 meningioma. (C) Routine surveillance imaging 15 months after surgery reveals tumor recurrence.

We assessed associations between preoperative telomerase levels and study variables, as shown on Table 2 for meningiomas and on Table 3 for GBM. For the meningioma cohort (Table 2) the mean preoperative telomerase transcript level differed significantly between patients without tumor recurrence (2.17 ± 11.7) and those with subsequent tumor recurrence (3.59 ± 4.42; *p* = 0.002). These data relate to patients with a minimum follow‐up of 12 months and it suggests that high telomerase transcript levels in blood‐derived exosomes prior to surgery likely indicate that the patient may be at high risk for tumor recurrence. This is possibly true for both WHO 1 and WHO 2 meningiomas. Figure 3 demonstrates one such example for WHO 1 meningioma. For the remaining 27 (23.1%) the data regarding tumor recurrence are not available either due to missing data or due to short surveillance period. Of note, their telomerase levels (0.56 ± 0.81) were within the normal range and did not differ significantly from those of healthy controls. No significant correlations were found between telomerase levels and all the other clinical variables that are presented on Table 2.

**FIGURE 4:**
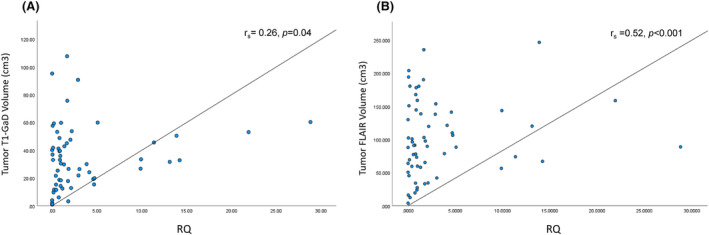
Association between the preoperative tumor volume and the serum relative quantification (RQ) for telomerase expression (A). Association between the enhancing tumor volume measured on preoperative gadolinium enhanced T1‐weighted images and the preoperative serum RQ levels. (B) Association between preoperative FLAIR volume and serum RQ levels prior to surgery.

Clinical data and hTERT transcript levels for the GBM cohort are presented in Tables 1 and 3. Values in the GBM samples were significantly different when compared to healthy controls, both the mean telomerase transcript levels (4.85 ± 11.97 vs. 0.5 ± 0.67) and the proportion of hTERT cases with above normal cutoff values (36% vs. 2.4%; *p* < 0.001). No association was found between preoperative telomerase transcript level and the clinical variables that are specified in Table 3. However, there was a positive correlation between the enhancing tumor volume and the pre‐operative transcript level (*r*_*s*_ = 0.26, *p* = 0.04). Likewise, the levels of exosomal hTERT transcript positively correlate with tumor FLAIR volume (*r*_*s*_ = 0.52, *p* < 0.001; Figure 4).

Longitudinal serum samples were available in the tumor biobank for GBM patients and served for evaluation of possible dynamics in the circulating exosomal hTERT transcript levels during the disease. The results for the four time points are presented in Table 3 and Figure 2B. We compared the mean transcript levels of three post‐operative time points to the baseline (the preoperative) samples (Table 3). It is notable that no significant change from preoperative level was observed after surgery and following the concurrent course of radiotherapy with temozolomide. However, the only close to significant difference was found between the preoperative mean level (4.51 ± 11.97) and the level detected following three adjuvant temozolomide cycles (2.17 ± 3.07) (*z* = −1.92, *p* = 0.06). Whether it suggests a maximal tumor response or clearance of exosomes from the circulation remains to be elucidated. Interestingly, as demonstrated on Figure 2B there was no decline in the proportion of elevated telomerase expression overtime for GBM cases. It remained significantly higher than the control group for all the timepoints that followed the surgical intervention (postoperative high expression rate‐50%; post radiotherapy‐66.6%; post three adjuvant temozolomide cycles‐47%; *p* < 0.01).

**FIGURE 5:**
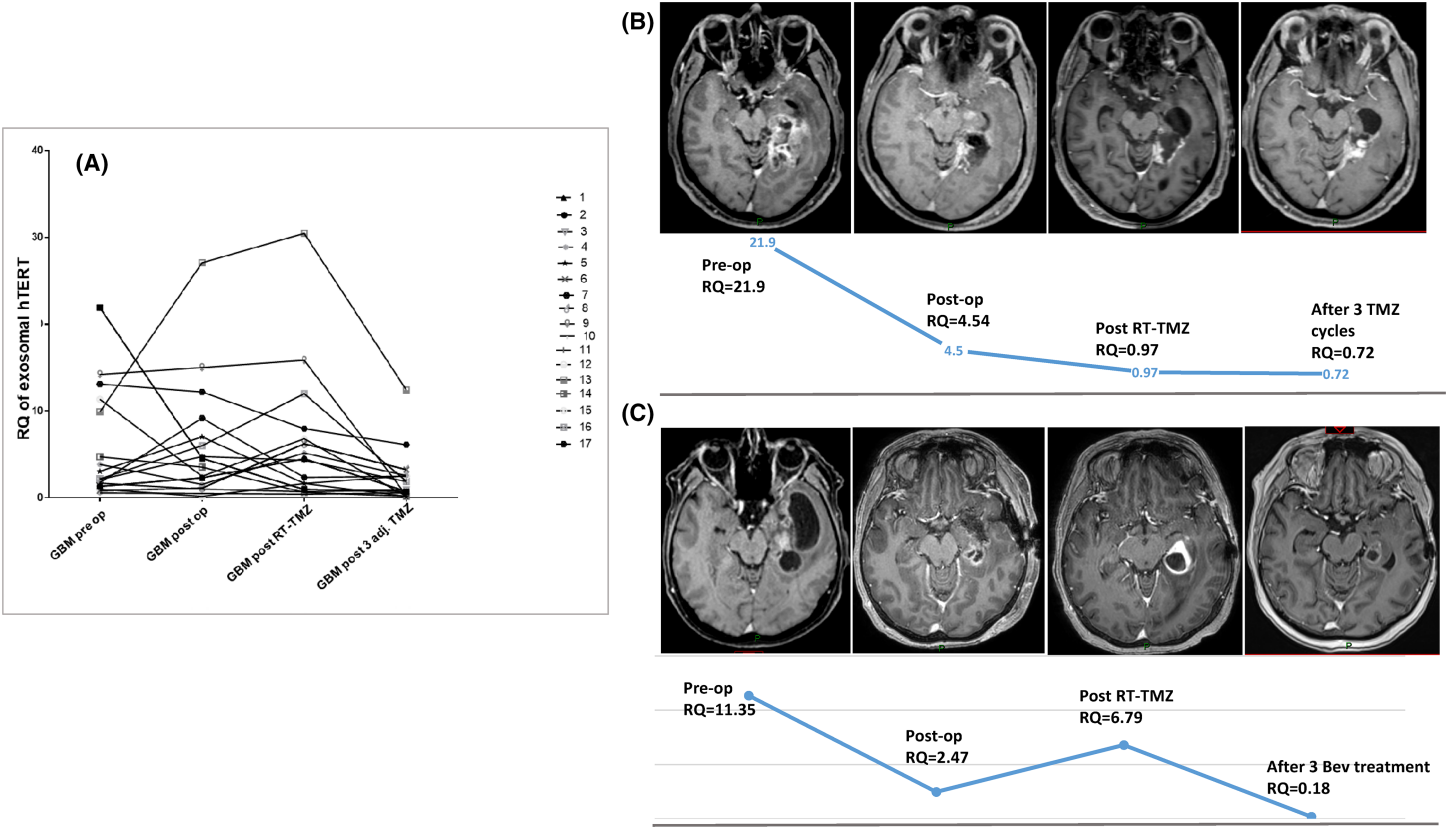
Fluctuations of hTERT transcript levels on longitudinal monitoring in glioblastoma patients. (A) The relative quantification (RQ) of 4 serial serum samples obtained from 17 glioblastoma patients at the four time points of the study. (B) The RQ of four serial serum samples of a glioblastoma patient plotted against his matched MRI studies at each time point. The preoperative RQ value is markedly elevated. It declined post operatively but remained above normal control levels. At the end of the concurrent chemoradiation course RQ value dropped to the normal range and remained low during adjuvant temozolomide treatment. MRI images show gradual collapse of the surgical bed without evidence for tumor recurrence. (C) The RQ of 4 serial serum samples of a glioblastoma patient plotted against his matched MRI studies at each time point. The preoperative RQ value is elevated. It declined post operatively but remained above normal control levels. At the end of the concurrent chemo‐radiation course MRI showed an enlarged enhancing mass and the RQ has scaled up. MR‐perfusion study (not shown) suggested tumor recurrence and the patients was put on biweekly bevacizumab treatment. Following three bevacizumab treatments marked tumor response is evident on MRI and the RQ value dropped to the normal control range.

Even though no significant changes were observed in the mean transcript levels during the observation, fluctuation in the telomerase transcript values in individual patients reflected the clinical course of the disease. Figure 5A demonstrates the fluctuations observed in the 17 patients with four sequential serum samples and Figure 5B exhibits two cases, whose serial serum telomerase levels are plotted against their matched MRI analyses at each time point. Overall, it indicates that an initial high transcript level corresponds to an increased oncogenic activity and a shift to low or normal‐range level probably indicates its inhibition. Therefore, circulating exosomal hTERT transcript levels may serve as a biomarker for those tumors with high telomerase expression at diagnosis, in conjunction with the currently available clinical parameters.

**FIGURE 6:**
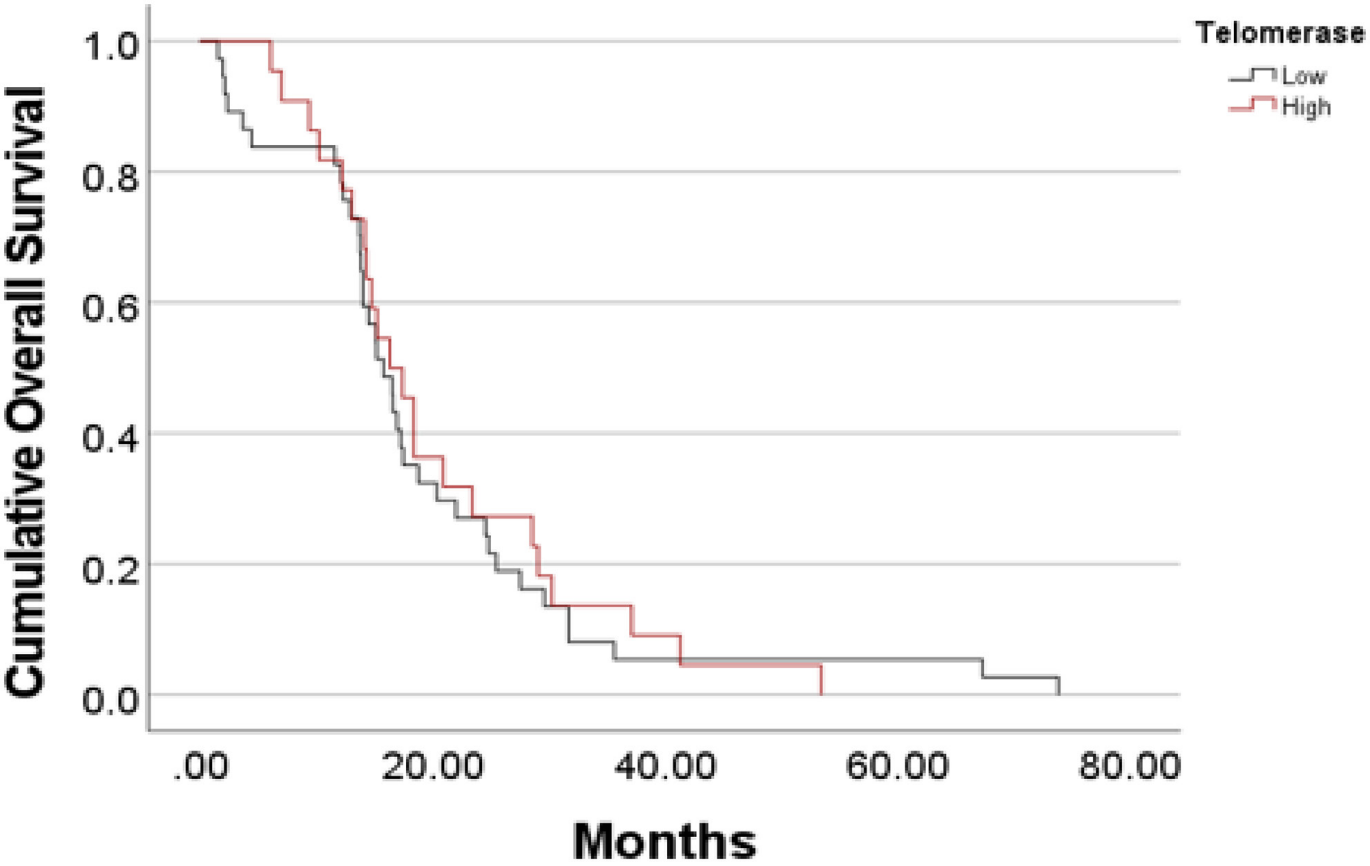
The Kaplan–Meier curves of overall survival of glioblastoma patients comparing survival of patients with elevated circulating preoperative hTERT transcript to the subgroup of patients with preoperative values within the healthy control range.

Even though GBM is a highly malignant tumor, high hTERT transcript levels were observed in only 36% of all initial GBM's serum samples. The mechanism and dynamics of exosomes release with high levels of hTERT transcript in some tumor is currently unknown. We explored whether there is a survival difference between those two GBM subgroups. Figure 6 shows the Kaplan–Meier survival curves of the two subgroups. No difference in survival patterns was observed (Log rank *p* = 0.745).

## 4 DISCUSSION

This study demonstrates, for the first time, that elevated blood derived exosomal hTERT transcript levels can be detected in serum of primary brain tumor patients. Our results suggest that the proportion of high levels of circulating telomerase transcript correlate with the extent of tumor malignancy and might serve as an indicator of oncogenic activity. These findings are in line with previous publications showing a similar phenomenon of increasing presence of circulating hTERT transcript with increasing tumor grade in both colorectal cancer^22^ and hepatocellular carcinoma (in preparations). Furthermore, our findings in meningiomas and glioblastomas suggest that it also has the potential to serve as a clinical biomarker. We demonstrated that high expression levels of exosomal hTERT transcript at diagnosis, correlated with later tumor recurrence in patients operated for WHO grade 1 and 2 meningiomas. As for glioblastomas, the exosomal transcript levels correlated with the volume of the enhancing tumor and reflected the clinical course of the disease for patients with high expression values at diagnosis.

Telomerase activity is upregulated in almost all neoplastic tissues^4^ including in primary brain tumors.^5^, ^23^ There are several known mechanisms that upregulate *TERT* transcription,^11^ but the one which has been largely investigated in both meningiomas and glioblastomas is related to *TERT* promoter mutation.^24^, ^25^, ^26^, ^27^, ^28^, ^29^, ^30^, ^31^ Analysis of meningioma tissues showed that hTERT promoter mutations occur in about 6%–8% of all meningiomas and rates increase with rising WHO grade. Hence, mutations are sparsely found in grade 1 tumors (1%–4.7%) but are described in 6%–7.9% and 14%–15.4% of WHO 2 and 3 meningiomas, respectively.^24^, ^26^ In comparison, we detected remarkably higher rates of elevated telomerase transcript values in the serum of both Grade 1 (16.25%) and Grade 2 (18.9%) tumors. These findings probably relate to the fact that telomerase expression is the final product of various mechanisms that induce upregulation of telomerase activity while TERT promoter mutation status represents only one of them. Conversely, in glioblastomas the rate of serum high telomerase expression level was notably lower (36%) from the rate of tumor TERT promoter mutations (79.6%). Based on the fact that TERT promoter mutations are the most frequent genomic alteration that occurs in 80%–90% of glioblastomas^31^, ^32^ it would be expected to find a higher rate of telomerase transcript in the circulation. However, several factors may have contributed to this discrepancy. One possible explanation for the lack of detectable circulating hTERT might be steroid treatment that interferes with the release of exosomes from tumor cells.^33^ Unfortunately, detailed information on pre‐operative steroid therapy (which is routinely used in GBM) was not always available for the study cohort. Another possible explanation is that exosomal hTERT transcript constitutes just a fraction of the total amount of the transcripts which are engulfed into extracellular vesicles (EV) by the GBM cells. These membrane vesicles are commonly classified as exosomes and microvesicles (MVs).^34^, ^35^ Exosomes, 30–150 nm in diameter, are packaged in the late endosome and are generated by the fusion of multivesicular bodies with the plasma membrane. MVs are usually defined as vesicles of 100–1000 nm that are budding/blebbing from the plasma membrane. Other types of EVs include ectosomes, membrane particles, exosome‐like vesicles, and apoptotic vesicles. All EVs are important regulators of cell–cell communications and can promote tumor progression and resistance mechanisms.^35^ Therefore, future studies should evaluate whether assessment of EV's transcript transcript level rather than just the exosomal fraction yields an improved reflection of telomerase expression in GBM. Furthermore, extraction of EVs from the cerebrospinal fluid (CSF) may increase the sensitivity of this analysis due to CSF proximity to the tumor and the presence of a direct exchange with brain parenchymal extracellular content. Finally, the content of exosomes is not merely a passive reflection of its constituents' cellular concentrations. The involvement of the endosomal sorting complex required for transport (ESCRT) complex in packaging nucleic acids in exosomes has been partially elucidated^36^ and it may differ in various patients, depending on the cancer type and the activity of the protein members of the ESCRT complex.

About 20% of meningiomas display aggressive behavior with early recurrence.^37^ Pathological grading of meningiomas cannot reliably predict the tumor behavior and clinical course as it provides no information concerning molecular alterations indicative of tumor aggressiveness. A recent study showed that methylation profiles, mutations and copy number variations are reflected in EV‐DNA of meningiomas.^20^ We showed that high exosomal hTERT transcript levels at initial diagnosis correlated with tumor recurrence. This was true for both WHO grade 1 and grade 2 meningiomas and indicates that the findings presented, could to be utilized for detection of tumors with a higher risk of early recurrence. This liquid biopsy technology is relatively simple and therefore, the turnaround time from sampling‐to results is short (up to 2 days). As such, it is a low‐cost analysis that can be easily implemented for a routine use. However, our exploratory results still need to be validated in future larger clinical studies as the current study has several limitations. The limitations include the retrospective collection of clinical data, which may have an inherent bias. Also, the cellular telomerase activity in tumoral tissues was not evaluated, thus we could not correlate between tumor and exosomal hTERT. In addition, the exosomes isolated from serum samples may have suffer from influences from platelet‐derived particles which may have affected the results. Lastly, we had a small number of longitudinal samplings to allow for clear‐cut evaluation of the test merit as a circulating biomarker.

In conclusion, our study demonstrated for the first time that the expression of hTERT transcript can be measured in the serum of both meningioma and glioma patients. We also showed that the rate of detection increased with rising tumor grade. As it correlated with tumor recurrence in meningiomas and with tumor volume and the disease course in glioblastomas it carries the potential to serve as a biomarker once used in conjunction with other clinical and radiological parameters. Future studies are necessary to investigate whether the sensitivity could be augmented and whether it can be implemented into routine patients care.

## AUTHOR CONTRIBUTIONS

**Orit Uziel:** Conceptualization (equal); investigation (equal); resources (equal); supervision (equal); writing – original draft (equal); writing – review and editing (equal). **Andrew Kanner:** Resources (equal). **Einat Beery:** Data curation (equal); methodology (equal). **Sapir Lev:** Data curation (equal). **Meir Lahav:** Funding acquisition (equal). **Suzana Horn‐Fichman:** Investigation (equal). **Sagi Har Nof:** Software (equal). **Yuseph Laviv:** Formal analysis (equal). **S. Yust‐Katz:** Visualization (equal). **Alexandra Amiel:** Formal analysis (equal). **Ramez Abu Shkara:** Methodology (equal). **mustafa siddeeq:** Methodology (equal). **Adva Levy‐Barda:** Methodology (equal). **Pia Raanani:** Resources (equal). **yaron Sela:** Software (equal). **Zvi R. Cohen:** Resources (equal). **Tali Siegal:** Conceptualization (equal); funding acquisition (equal); investigation (equal); resources (equal); writing – original draft (equal); writing – review and editing (equal).

## Data Availability

All the data relevant to this manuscript was submitted and is therefore available to all.
